# Desmoplasia and Chemoresistance in Pancreatic Cancer

**DOI:** 10.3390/cancers6042137

**Published:** 2014-10-21

**Authors:** Marvin Schober, Ralf Jesenofsky, Ralf Faissner, Cornelius Weidenauer, Wolfgang Hagmann, Patrick Michl, Rainer L. Heuchel, Stephan L. Haas, J.-Matthias Löhr

**Affiliations:** 1 Division of Gastroenterology, Endocrinology and Metabolism, University Hospital, Philipps-Universitaet Marburg, Baldingerstrasse, Marburg 35043, Germany; E-Mails: Marvin.Schober@med.uni-marburg.de (M.S.); michlp@med.uni-marburg.de (P.M.); 2 Department of Medicine II (Department of Gastroenterology, Hepatology, and Infectious Diseases), University Medical Center Mannheim (UMM), Theodor-Kutzer-Ufer 1-3, Mannheim 68135, Germany; E-Mails: ralf.jesenofsky@medma.uni-heidelberg.de (R.J.); ralf.faissner@web.de (R.F.); C_Weidenauer2000@yahoo.de (C.W.); 3 Lung Cancer, Genomics/Epigenomics Group, Division of Epigenomics and Cancer Risk Factors, German Cancer Research Center (DKFZ), Im Neuenheimer Feld 280, Heidelberg 69121, Germany; E-Mail: w.hagmann@dkfz-heidelberg.de; 4 Department of Clinical Science, Intervention and Technology (CLINTEC), Karolinska Institutet, SE-141 52 Huddinge, Sweden; E-Mail: rainer.heuchel@ki.se; 5 Gastrocentrum, Karolinska University Hospital, Stockholm, Stockholm 141 86, Sweden; E-Mail: stephan.haas@karolinska.se

**Keywords:** pancreatic cancer, desmoplasia, drug resistance, SFM-DR, CAM-DR, *de novo* resistance, cancer stem cells, matrix, future therapies

## Abstract

Pancreatic ductal adenocarcinoma (PDAC) occurs mainly in people older than 50 years of age. Although great strides have been taken in treating PDAC over the past decades its incidence nearly equals its mortality rate and it was quoted as the 4th leading cause of cancer deaths in the U.S. in 2012. This review aims to focus on research models and scientific developments that help to explain the extraordinary resistance of PDAC towards current therapeutic regimens. Furthermore, it highlights the main features of drug resistance including mechanisms promoted by cancer cells or cancer stem cells (CSCs), as well as stromal cells, and the acellular components surrounding the tumor cells—known as peritumoral desmoplasia—that affects intra-tumoral drug delivery. Finally, therapeutic concepts and avenues for future research are suggested, based on the topics discussed.

## 1. Introduction

In 2012, 43,920 cases of pancreatic cancer were reported in the US, and the disease ranked 10th in the annual statistics of newly diagnosed carcinomas. However it was quoted as the 4th leading cause of cancer deaths and is projected to be the 2nd biggest cancer killer in the United States by 2030 [1,2]. A median survival time of only 6 months and a long-term survival rate of approximately 4% underscore the very aggressive behavior of this disease [3,4]. Pancreatic ductal adenocarcinoma (PDAC) is a disease of the over fifties [5]. In addition to smoking, germline mutations (with BRCA2 as the most frequent mutation), chronic pancreatitis and diabetes mellitus have all been described as risk factors for the development of PDAC [6]. Due to its long, asymptomatic clinical course with the first diagnosis being made usually at an advanced stage of the disease, only 15% of patients are eligible for surgical therapy, which still represents the only potentially curative treatment strategy [5]. However, PDAC’s aggressive and early metastatic behavior frequently prevents even curative resection. Even without metastases, local infiltration of retroperitoneal major vessels can exclude pancreatic resection. Thus curative resection is the exception rather than the rule and a curative non-surgical option is still not yet in sight [4].

Current chemotherapeutic regimes are restricted to neoadjuvant, adjuvant or palliative settings, mostly using gemcitabine-based chemotherapy protocols. Recently, a combination therapy using oxaliplatin, 5-FU, irinotecan and folinic acid (FOLFIRINOX) has shown significant survival benefits in patients with good performance status [7]. This palliative regimen is being evaluated in clinical trials, such as the current CONKO-007 Study (EUDRACT Nr. 2009-014476-21) amongst others, as an adjuvant or neoadjuvant concept [8,9,10,11]. Targeted therapies have mostly shown disappointing results in pancreatic cancer until erlotinib, an epidermal growth factor receptor (EGFR) tyrosine kinase inhibitor, in combination with gemcitabine, became the first treatment-additive to show a very moderate, but significant survival advantage. Consequently, it has been approved for the treatment of PDAC [12,13,14,15,16].

Recently compounds targeting components of the desmoplastic stroma of the tumors, for example, endothelial cells and pancreatic stellate cells (PSCs) have shown beneficial effects in preclinical models and in clinical trials. The targeted treatment of tumor endothelial cells using the anti-microtubule agent, paclitaxel, embedded in cationic liposomes (EndoTAG-1) is reported to be generally well tolerated and has shown beneficial survival rates in a phase II trial, resulting in disease control-rates of between 52% and 65%, and a median overall survival ranging from 8.1 to 9.3 months [16]. Comparable results were obtained with albumin-bound paclitaxel (nab-paclitaxel [Abraxane^®^]), achieving disease control-rates of 67% and an overall survival of 9 months when combined with gemcitabine in several trials [17,18,19]. The US Food and Drug Administration (FDA) has now approved this compound for clinical use in late-stage PDAC. Here osteonectin (also known as SPARC), which is highly expressed and secreted by peritumoral pancreatic stellate cells, might serve as an albumin binding protein facilitating intra-tumoral drug accumulation. However, genetically engineered mouse models of pancreatic carcinoma showed comparable nab-paclitaxel concentrations in tumors in SPARC null mice thus questioning the proposed mechanism of drug accumulation [20]. EndoTAG-1 and nab-paclitaxel are examples of the concept of increasing anti-tumorigenic efficacy by combined targeting of the microenvironment as well as neoplastic cells in an attempt to overcome the chemoresistance of PDAC [16,17,18,19].

## 2. Concepts of Therapy Resistance

**Figure 1:**
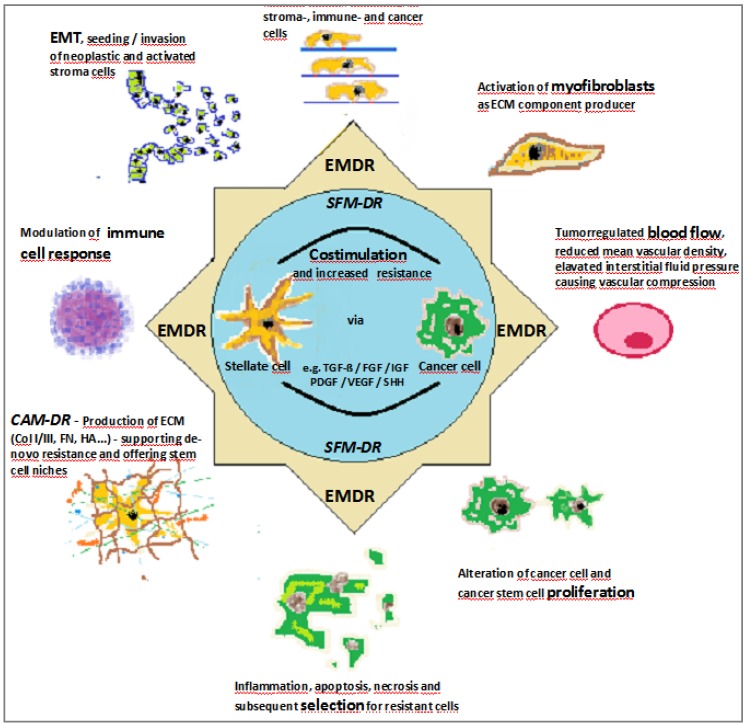
Octagon of environmental mediated drug resistance (EMDR) in pancreatic cancer. Soluble factor mediated drug resistance (SFM-DR), a form of EMDR (yellow), is shown in blue.

An ever-increasing number of factors contributing to chemoresistance have been identified in recent years, while it is also well recognized that the tumor microenvironment and the cross-talk between different cell populations residing in this milieu are major contributors to the resistance observed in many tumors. The tumor microenvironment of PDAC consists of distinct cell types including endothelial cells, immune cells, fibroblasts, endocrine cells and, in particular, PSCs embedded in a matrix of extracellular proteins (Figure 1) [21].

Many investigators have highlighted the fact that impaired drug delivery is due to the characteristically dense desmoplastic reaction in PDAC [14,22,23,24]. In addition, several reports have demonstrated that the differential expression of multidrug resistance-associated proteins (MRPs) exists as a resistance mechanism in cancer cells [25,26,27]. The principles of resistance listed below reflect the complexity of the mechanisms contributing to chemoresistance and, furthermore, have to be classified as an either *de novo* or acquired resistance phenotype (Figure 1).

This review focuses on models that helped to explain the mechanisms underlying chemoresistance in PDAC. Based on recent research, four main resistance principles are highlighted:

1. Cancer cells or cancer stem cells (CSCs) promote resistance mechanisms
2. Stroma cells promote resistance via establishment of the desmoplastic microenvironment
3. Matrix components increase drug resistance
4. High interstitial pressure prevents intra-tumoral drug deposition

### 2.1. Identifying Resistance Mechanisms of Cancer Cells or CSCs

Various different cancer cell mechanisms have been characterized to explain drug-mediated apoptosis. Overexpression of transporter proteins, such as MRPs and MDR1/P-glycoprotein, in various tumors and cell lines displaying a resistance phenotype has been known for 30 years [28,29,30,31,32,33,34,35]. However, in PDAC, the relevance of these transporter proteins has long been unclear. In 2009, our group verified that treatment with 5-fluorouracil (5-FU) and/or gemcitabine induced an up-regulation of MRP-transporters in PDAC tumor cells [36]. We demonstrated an elevated expression of MRP3, MRP4 and MRP5 in a 5-FU-resistant Capan-1 cell line (adapted to 2 µg/mL 5-FU via gradual exposure to 5-FU in the culture medium). MRP5, in particular, contributed to 5-FU resistance as the knock-down of MRP5 mRNA resulted in an increased sensitivity towards 5-FU treatment [36]. The same effect was seen in Patu-02 cells, as reported by Nambaru and colleagues [37]. Extending our findings, we demonstrated that MRP-5 also contributed to an increased resistance towards gemcitabine in PANC-1 cells. Again, silencing of MRP5 sensitized PANC-1 cells to gemcitabine treatment. This result clearly showed that altered transporter expression is important in overcoming the toxic effects of prolonged drug treatment [38].

Interestingly, in 2007, Shah and colleagues reported that an epithelial-mesenchymal-transition (EMT) is linked to gemcitabine resistance in pancreatic carcinoma cells. EMT was found to be associated with the activation of c-Met upstream of PI3 kinase, thus mediating NF-kB and AKT activity [39]. Both of them are known to be regulators of apoptosis or cell survival. This observation suggested that the altered expression levels of ABC transporters might also be induced via these pathways [36,38,40].

A broad-spectrum genetic analysis by Jones and colleagues covering 24 pancreatic cancer samples revealed an average of 63 genetic alterations in every case of PDAC. These alterations affected 12 overlapping core signaling pathways that were modified in 67%–100% of the PDAC samples analyzed [41]. It is worth noting that a relatively large number of genetic alterations in PDAC affected only a small number of core pathways. Therefore, a therapy targeting nodal points or redundant key regulators in these altered pathways would seem to be more promising than targeting single mutated genes. Obviously, conclusions regarding key regulators cannot be drawn from these data; moreover, since the hallmarks of cancer are known to be regulated by redundant pathways, the targeted treatment of a single cancer cell pathway may stimulate the activation of alternative redundant signaling pathways, enabling cancer cell survival by other means [42,43].

Another cause of treatment failure is the inability of the treatment to eliminate the population of tumor stem cells. Whereas the bulk of the tumor mass can be reduced successfully via cytotoxic treatment, CSCs (characterized by a high intrinsic resistance to chemo- and radio-therapy) will survive and give rise to a new generation of cancer cells that are resistant towards the applied chemotherapeutics. Interestingly, several groups have also reported that the process of undergoing an EMT leads to an increase in the number of cells exhibiting stem cell-like features, and that this transdifferentiation process itself increases resistance towards chemotherapeutics [39,44,45,46,47,48,49]. In addition, co-culture experiments performed by Gupta and colleagues showed that when telomerase-immortalized human mammary epithelial (HMLE) cell subgroups were treated with paclitaxel, finally those cells survived that had undergone EMT. Hence, these experiments illustrate that the differentiation state of the targeted cell population is also of importance and might demand a more individualized approach to the treatment of cancer [47].

The importance of the surrounding microenvironment for the control of CSC self-renewal and differentiation has recently been expanded to include PSCs [50,51]. These cells offer a microarchitectural stem cell niche and support the self-renewal and invasive properties of CSCs. PSCs and CSCs overexpress the TGF-ß superfamily members, nodal and activin [50], the importance of which for tumorigenesis and invasion has already been demonstrated in various types of tumors such as esophageal cancer and melanoma [22,52,53,54]. As shown by Lonardo and colleagues for PDAC, interfering with nodal signaling *in vitro* decreased the self-renewal and sphere formation of CSCs; however, inhibition of tumorigenesis *in vivo*, has only been sufficient when gemcitabine was added while nodal signaling was abrogated [50]. With respect to the CSC population in PDAC, its precise origin as well as its relationship to EMT still requires clearer definition, although the link between CSCs and drug resistance seems clear.

### 2.2. Stromal Cells Promote Resistance via Establishment of the Desmoplastic Microenvironment

The pancreatic stellate cell (PSC) is an important stromal cell type in PDAC. In the normal pancreas PSCs are located in the periacinar space and account for about 4% of all pancreatic cells. These PSCs are quiescent (non-activated) and are characterized by cytoplasmic lipid droplets containing vitamin A. After activation in response to cytokines, oxidative stress, or other factors, PSCs start to proliferate and to produce cytokines and large amounts of extracellular matrix (ECM)-components including laminin, desmin, and collagen I/III (Col I/III) until they eventually assume a myofibroblast-like phenotype [14,26,55]. Activated PSCs are characterized as major drivers of cytokine and ECM production, finally culminating in the typical hypovascular and fibrotic microenvironment found in chronic pancreatitis and PDAC [14,26,27]. Our group has successfully established immortalized PSCs by transfection of PSCs (grown out from tissue obtained from a patient with chronic pancreatitis) with SV40 large T antigen and human telomerase reverse transcriptase (hTERT). The immortalized cells retained characteristics of the native activated PSCs, including expression of vimentin, desmin, alpha smooth muscle actin (αSMA) and glial fibrillary acidic protein (GFAP). Moreover they responded to physiological stimuli in the same way as their native counterparts by increased proliferation after platelet-derived growth factor (PDGF) stimulation, and with increased expression of transforming growth factor ß1 (TGFß1) and ECM proteins such as Col I and fibronectin (FN) upon TGFß1 stimulation [26]. The autocrine TGFß1 loop may be of particular relevance with respect to perpetuating the fibrotic process initiated by external stimuli [26,56].

In 2001, our group presented *in vivo* data illustrating the induction of stroma in nude mice after orthotopical injection of PANC-1 cells stably transfected with TGFß1 cDNA. As no stromal cells were co-injected, this process relied upon the stimulation of host stromal cells adjacent to the injected cancer cells via TGFß1 signaling. Thus, the impact of TGFß1 on stromal induction was demonstrated *in vivo* using transfected cancer cells as the primary producers of TGFß1 [57]. More recently, Apte and Wilson observed the activation of stellate cells by neoplastic cells or their culture supernatant, confirming the interaction between PSCs and pancreatic cancer cells (Figure 1) [58].

Another factor contributing to chemoresistance is the induction of EMT in cancer cells by PSCs. In cancer cells, EMT is characterized by decreased expression of epithelial markers, such as e-cadherin, and increased levels of mesenchymal markers, including vimentin, and is associated with a decrease in drug susceptibility [39,49]. The co-culture of cancer cells and PSCs induced an alteration in expression of the CSC-related genes: nestin, ABCG2 and LIN28 in the cancer cells, indicating a CSC-like phenotype. These results emphasize an active role for PSCs in initiating cancer cell EMT as a concomitant feature of resistance [39,47,59], and vice versa since PDAC cells were shown to stimulate PSC activation and proliferation [60]. The subcutaneous injection of pancreatic cancer cells with, and without, PSCs into nude mice resulted in larger tumors when PSCs were co-injected. Interestingly, morphometric analysis revealed that this was not only the result of the fibrotic reaction mediated by PSCs but was also due to increased cancer cell proliferation [61,62]. In an orthotopic mouse model, in addition to increased tumor growth, enhanced metastasis was also observed after co-injection of pancreatic tumor cells and PSCs. In these tumors, the researchers observed bands of fibrosis and αSMA-positive, activated PSCs that were associated with increased tumor cell proliferation and reduced apoptosis. In cases of metastatic liver nodules, histomorphological analysis revealed αSMA-positive cells that also stained positive for human nuclear antigen, indicating that PSCs are able to co-metastasize with cancer cells, thus providing a niche for cancer cell survival and proliferation at distant sites [63,64]. *In vitro*, PSC migration was increased in the presence of pancreatic cancer cells, while PSC secretions reduced apoptosis and increased the proliferation and migration of cancer cells. The use of antibodies to neutralize PDGF in the conditioned media inhibited the proliferation of tumor cells [64]. With respect to the model of environment-mediated drug resistance (EMDR), discussed in the next chapter, the observed repression of apoptosis in the presence of pancreatic stellate cells fits quite well and may partly be induced by proteins like FN and cytokines secreted by the PSCs [14,25,64,65,66,67,68].

Targeting the tumor stroma using a sonic hedgehog (SHH) inhibitor in combination with gemcitabine in the KPC mouse model (a transgenic expression model of PDAC) resulted in elevated gemcitabine levels in the tumors and increased cancer cell destruction. This effect has to be regarded as a consequence of the architectural changes in the tumor microenvironment [22]. However, keeping in mind that PSCs might supply a stem cell niche for CSCs (see above), gemcitabine treatment may only reduce the bulk tumor mass while sparing the residing CSCs, with the resulting risk of tumor recurrence [50]. Thus it is clear (from the above) that both tumor cells and stromal cells have to be targeted for the development of promising therapies for PDAC.

### 2.3. Matrix Components Increase Drug Resistance

In 1999, Damiano and colleagues established the concept of cell adhesion-mediated drug resistance (CAM-DR), a *de novo* drug-resistant phenotype, in multiple myeloma. Myeloma cells, primarily susceptible to melphalan and doxorubicin, survived short-term treatment as well as serum starvation when bound to ECM components, such as FN, via the integrins α4β1 or α5β1 [66]. Strikingly, these cells showed neither a reduced intracellular drug concentration nor higher levels of BCL-2 anti-apoptotic family members such as BCL-2 or BCL-xl. This effect was reversible and cells reverted to a chemosensitive phenotype comparable to their susceptible parental cells. The progression of apoptosis in cells not pre-adhered to FN, when treated with melphalan and doxorubicin, could not be arrested when the cells were exposed subsequently to FN [66]. In 2000, Hazlehurst and colleagues undertook a comparison of genomic changes in *de novo* resistant multiple myeloma (CAM-DR) cells *versus* their corresponding acquired resistance cell line that revealed a total of 1479 altered genes in the latter group compared to 69 in the CAM-DR myeloma cells [67,68]. Furthermore, it was demonstrated that increased levels of P27kip1, associated with adhesion to FN, are related to the CAM-DR phenotype. Consequently, treatment with P27kip1 antisense oligonucleotides induced a partial drug response without compromising the FN adhesion [67]. Topoisomerase II and the anti-apoptotic regulator, c-FlipL, are further targets for cancer therapies, since they have been linked to the CAM-DR phenotype and are modified by cellular adhesion. However, it should be considered that CAM-DR might also represent a redundant survival pathway as well as a β1-Integrin-mediated resistance mechanism. Several other groups have shown that a variety of expressed integrins are linked to growth promoting effects via paracrine and autocrine signaling pathways [69,70,71,72,73], and this should be taken into account when attempting to revert a CAM-DR phenotype *in vivo* [25]. As mentioned above, Damiano and Hazlehurst were the first to demonstrate a conclusive model for *de novo* chemoresistance in initially drug-susceptible cancer cells. This model revealed that cellular adhesion provides a distinct survival advantage to initial drug treatment and facilitates subsequent transformation and selection for acquired resistance mechanisms via stepwise genetic adaption.

Although established for multiple myeloma, the CAM-DR concept was later extended to a variety of solid tumors [74,75,76]. In PDAC, the adhesion of tumor cells to ECM proteins also conferred resistance to chemotherapy *in vitro* [77,78], while a meta-analysis indicated that CAM-DR may occur in PDAC since genes for proteins that are expected to be linked with CAM-DR, such as the integrins and members of the PI3K-mediated signaling pathways, are frequently over expressed [79]. More than 30 years ago, an increase in the resistance of tumor cells to cytotoxic drugs was observed when the tumor cells were cultured as 3-dimensional (3D) spheroids rather than the normal 2D monolayer in *in vitro* cultivation [80,81,82]. This effect was subsequently termed multicellular resistance. In the years following, various factors contributing to this multicellular resistance have been described including reduced drug uptake, reduced proliferation and altered gene expression [83,84,85]. Disrupting cell contacts with hyaluronidase reversed the resistance induced by 3D culture as did interfering with integrin signaling or the inhibition of focal adhesion kinase (FAK), demonstrating that cell-cell or cell-matrix interaction are also important in this model system [86,87,88], making it an ideal tool with which to analyze drug resistance mechanisms.

Recently, our group extended the knowledge of CAM-DR with regard to PDAC using 3D spheroids of several human pancreatic tumor cell lines, and cell lines derived from the KPC mouse model of PDAC. We established that PDAC cancer cells grown in spheroids were also more resistant to cytotoxic drugs than cells cultivated in 2D tissue culture. Additionally, we found that the synthesis of matrix proteins as well as expression of the stromal markers lumican, SNED1, DARP32 and miR-146a, increased significantly in 3D spheroids [89].

With respect to resistance-promoting ECM synthesis, as well as pro-survival or proliferative signaling cascades, soluble mediators involved in soluble factor mediated drug resistance (SFM-DR) should also be investigated. In particular, the relevance of TGFß1 should be addressed once again. Our previously published data concerning the over-expression of TGFß1 in PANC-1 cells revealed a positive correlation between stroma induction and TGFß1 expression. Co-cultivation *in vitro* induced an up-regulation of matrix proteins and growth factors in both the tumor cells and the co-cultivated fibroblasts with a concomitant increase in proliferation of both cell lines. *In vivo* orthotopical transplantation of TGFß1-over-expressing PANC-1 cells into nude mice induced the abundant stroma characteristic of PDAC, an effect that can be attributed to the up-regulation of matrix proteins (Col I, Col III, FN *etc.*) and growth factors (TGFß1, PDGF, fibroblast growth factor [FGF], connective tissue growth factor [CTGF] *etc.*) mentioned above [57,64]. These divergent mediators may be regarded as forming the fundamental background of SFM-DR in pancreatic carcinoma. The conclusive data of Bachem and colleagues regarding ECM induction by pancreatic cancer cells and PSCs lays particular stress on the relevance of TGFß1 and FGF and highlights both cell types as co-stimulatory drivers of SFM-DR [61].

### 2.4. Tumor Perfusion to Increase Drug Accumulation

It has been reported that PDACs are rather poorly perfused compared to inflammatory pancreatic processes or other tumor types, especially endocrine tumors [90,91,92]. Comparing tumors induced via transgenic expression in a KPC mouse model of PDAC with orthotopically implanted pancreatic tumors, the former show a dysfunctional vasculature contributing to an impaired drug delivery and increased resistance to gemcitabine treatment. In contrast, orthotopically implanted pancreatic tumors demonstrate a functional vasculature and respond to gemcitabine. The active intracellular gemcitabine metabolite (2',2'-difluorodeoxycytidine triphosphate [dFdCTP]) was undetectable in tumors from KPC mice while high levels were revealed in xenografted tumors, thus dFdCTP accumulation correlated strongly with the responsiveness to drug treatment [22].

Various groups have demonstrated the importance of SHH signaling for the development and maintenance of the stromal compartment [93,94]. It seems reasonable to assume that interfering with this signaling cascade will deplete the PDAC stromal reaction since several groups have reported that SHH signaling is dysregulated in neoplastic cells of KPC mice and promotes desmoplasia at a level concordant to that seen in human pancreatic tumors [93,94,95,96]. Indeed, KPC mice treated with the Smo inhibitor, IPI-926, or a combination of IPI-926 and gemcitabine, demonstrated depletion of the stroma in pancreatic tumors. In morphologically ductal tumors, cells in treated tumors were arranged densely and tumors had a higher mean vascular density (MVD), while Col I levels were decreased compared to control mice treated with vehicle or gemcitabine alone. Consequently, the higher MVD led to an optimized drug delivery in IPI-926-treated mice, and significantly prolonged survival and decreased metastasis to the liver in the IPI-926-plus-gemcitabine treatment group. However, these effects were only transient; after a decrease in tumor size within the first two weeks of treatment, tumor growth resumed and the MVD dropped, suggesting that the tumor had adapted to the drugs [22]. These impressive results illustrated that targeting the stroma increased treatment efficiency by augmenting local drug concentrations, whereas the value of anti-angiogenic molecules, such as vascular endothelial growth factor (VEGF), might be overestimated in this particular tumor environment. Later, two groups extended these findings using the same mouse model. Tumors in KPC mice are comparable to human PDAC tumors and show a high level of the glycosaminoglycan, hyaluronic acid (HA). Injection of pegylated recombinant human hyaluronidase PH20 (PEGPH20), into tumor-bearing KPC mice resulted in the rapid degradation of HA in the tumors, which restored the patency of intra-tumoral vessels and increased vessel diameters. Morphologically, this process was accompanied by fenestrations and inter-endothelial junctional gaps in the endothelia of PDAC tumors. Remarkably, normal vasculature did not show these morphological changes after PEGPH20 treatment. Moreover, the high interstitial fluid pressure within the tumors was reduced to normal levels. These alterations resulted in a tumor-specific increase in permeability for macromolecules, and combined treatment with gemcitabine and PEGPH20 dramatically improved treatment efficacy resulting in a near doubling of median survival time and a marked decrease in the metastatic burden [14,22,23,24].

Referring again to the reported data of Lonardo and colleagues, the pharmaceutical knockdown of the Alk 4/5/7 receptor (involved in nodal/activin signaling) by SB431542 resulted in a loss of the self renewal capacity of CSCs, and a resensitization to the gemcitabine treatment of orthotopic tumors obtained by injection of a single-cell suspension of CSCs *in vivo* with 100% survival of the treated mice within the follow up period. In contrast, this combination was insufficient to treat xenografted primary pancreatic cancer tissue successfully as tumor growth was not slowed down. Here again, the stroma-rich tissue of the primary tumors precluded drug delivery as shown by mass-spectrometry. Targeting the stroma by adding the SHH inhibitor, CUR199691, to the treatment regimen increased drug delivery 10-fold, prolonged progression free survival of the treated mice, and depleted the CSC subpopulation in the tumor remnants [50].

## 3. Conclusions and Future Perspectives

Since PDAC-related deaths are projected to become the 2nd leading cause of cancer deaths within the next decade or so, a multimodal approach, involving concomitant targeting of the stroma and neoplastic cells, is pivotal to realizing a therapeutic breakthrough for this almost uniformly chemoresistant cancer type [2]. Since stroma production is triggered and maintained by the activation of multiple cellular pathways and growth factors (e.g., TGFß1, hepatocyte growth factor [HGF], insulin-like growth factor [IGF], epidermal growth factor [EGF], FGFs *etc.*), these factors and their mediators represent a promising target for cancer therapy, which is in line with the concept of EMDR [14,97]. Consequently, a multitude of current clinical trials for PDAC involve targeting either one or several of these aberrantly activated signaling pathways, including SHH, Notch, TGFß1, FGF and PDGF, with conventional cytotoxic drugs. Addressing the pathomorphological features of PDAC, as reported in the nabPaclitaxel or EndoTAG clinical trials, will enhance the armamentarium of drugs available for treating this disease in a more adequate manner. In this context, initial data presented by Löhr and Jesenofsky with respect to the key role of PSCs as drivers for the desmoplastic stromal reaction demonstrated a close interaction and even co-stimulation between orthotopically injected cancer cells and surrounding stromal cells (Figure 1). Moreover, in addition to Jacobetz’s group, Olive and colleagues showed that the targeted treatment of stromal cells led to increased drug delivery and accumulation in the tumor, with a resultant increase in therapeutic efficacy towards the tumor cells themselves. The reported decreased vascularization of the tumors in these models explained conclusively why anti-VEGF therapies are most likely to fail in pancreatic cancer [22,24,57]. Nowadays, there are means to measure tumor perfusion and oxygenation *in vivo* with novel imaging tools, as demonstrated recently by Gerling and colleagues that might exempli gratia help to evaluate therapeutic efficacy of stromal depletion [98].

The work of Xu, Vonlaufen and colleagues has established an even more active role for PSCs by demonstrating the co-migration of PSCs and tumor cells during invasion and metastasis [63]. This co-migration may facilitate distant metastasis as well as local re-seeding as both cell types form a metastatic unit, which provides a supportive niche for tumor cell growth.

The concept of interacting cancer and stromal cells, in addition to cancer and ECM interactions, explains *de novo* resistance prior to the subsequent acquisition of resistance mechanisms, which require more complex genetic and epigenetic alterations. These interactions illustrate how—as well as a minor population of primarily resistant, or quickly adaptive CSCs—the bulk of neoplastic cells might be able to escape therapy-induced apoptosis. Therefore, focusing either on extracellular ligand-receptor interactions, down-stream pathways in tumor cells, or downstream pathways in tumor stroma, as well as targeting ECM components themselves, may represent the most promising strategy for future therapies [65].

Recently, Rhim and colleagues used a modified KPC mouse model to demonstrate that EMT occurs well before frank tumor development in pancreatic intraepithelial neoplasia 2 (PanIN-2) lesions. Cells from the PanIN-2 lesions, having undergone EMT, were readily detectable in the circulation and seeding of single cells into the liver was observable. These circulating PanIN-2-derived cells were enriched 100-fold for the cancer stem cell markers, CD44 and CD24, compared to the source pancreas and gave rise to tumor development after injection into NOD/SCID mice. Inflammation promoted EMT, invasion and dissemination of the PanIN-2-derived circulating cells, again proving the importance of inflammation in the process of PDAC development. Dexamethasone treatment almost eliminated the PanIN-2 lesions and markedly reduced the number of circulating PanIN-2-derived cells, which may avoid progression to an evident pancreatic ductal adenocarcinoma [99]. Kleeff and colleagues reported a similar observation of an activated stroma around PanIN-1 lesions, which was confirmed as absent around normal pancreatic ducts [21]. If we expect this to happen in humans as well it might necessitate a prophylactic treatment with anti-inflammatory drugs to circumvent this early seeding.
